# Evaluation of the Efficacy and Safety of Chinese Herbal Injection Combined With Trimetazidine for Viral Myocarditis: A Network Meta-Analysis

**DOI:** 10.3389/fphar.2021.630896

**Published:** 2021-04-29

**Authors:** Kerui Wu, Dingwei Deng, Binghui Yu, Ziyun Han, Lanlin Huang, Yaxing He, Xia Yan, Dawei Wang

**Affiliations:** ^1^ The Second Clinical College of Guangzhou University of Chinese Medicine, Guangzhou, China; ^2^ The Second Affiliated Hospital of Guangzhou University of Chinese Medicine, Guangdong Provincial Hospital of Chinese Medicine, Guangzhou, China; ^3^ Shunde Affiliated Hospital of Guangzhou University of Chinese Medicine, Shunde, China

**Keywords:** randomized controlled trials, network meta-analysis, viral myocarditis, trimetazidine, Chinese herbal injection

## Abstract

**Background:** Viral myocarditis (VMC) is a common emergency of cardiovascular disease. Current treatment for VMC includes the prohibition of exercise plus supportive and symptomatic treatment, given the lack of specific antiviral therapeutic options and insufficient evidence for the use of novel immunosuppressive therapies. Trimetazidine, a drug used to improve myocardial energy metabolism, is frequently used for the treatment of viral myocarditis. In China, Chinese herbal injections (CHIs) are often used in combination with trimetazidine. Therefore, we evaluate the efficacy and safety of CHI combined with trimetazidine in the treatment of VMC through the method of network meta-analysis.

**Methods:** We searched PubMed, the Cochrane Library, Embase, China National Knowledge Infrastructure (CNKI), Wanfang Database, Chinese Scientific Journals Full-text Database (VIP), and China Biology Medicine Database (CBM) databases from inception to September 1, 2020, to identify eligible randomized controlled trials. The Cochrane risk of bias tool was used to assess the risk of bias among selected studies and the Stata 16.0 software was used to perform the network meta-analysis.

**Results:** A total of 29 studies were included, representing data from 2,687 patients. The effectiveness rate, level of myocardial injury marker, and the adverse reaction rate were evaluated. Compared with conventional treatment or conventional treatment combined with trimetazidine, CHIs combined with trimetazidine appeared to have a better therapeutic effect, with higher effectiveness rate and better reduction of the levels of creatine kinase, creatine kinase-MB, and lactate dehydrogenase. Based on surface under the cumulative ranking, Shenmai injection combined with trimetazidine appeared to be superior in terms of effective rate, while *Astragalus* injection or *Salviae miltiorrhizae* and *ligustrazine hydrochloride* injection combined with trimetazidine appeared most effective in reducing myocardial injury markers. There was no significant difference in safety between the interventions. However, a lack of safety monitoring in some selected studies meant that the safety of some interventions could not be fully evaluated.

**Conclusion:** CHIs combined with trimetazidine may have therapeutic value in the treatment of viral myocarditis, and Shenmai injection, *Astragalus* injection, and *Salviae miltiorrhizae* and *ligustrazine hydrochloride* injection may represent the most effective CHIs. Further clinical investigation is required to confirm these results.

## Introduction

Viral myocarditis (VMC) is a localized or diffuse myocardial inflammatory lesion caused by viral infection. Its clinical manifestations vary widely, from asymptomatic in mild cases to heart failure and even sudden cardiac death in severe cases. The most common manifestations are chest pain, heart failure, and fatigue dyspnea (Grün et al., 2012; Society of Cardiovascular Diseases, China Association of Chinese Medicine, 2020). Viral infection is believed to be the main cause of VMC, and the common viruses are coxsackievirus B, parvovirus B19, herpesvirus, and so on (Leone et al., 2019). Relevant epidemiological data show that the incidence of VMC is about 10–22 per 100,000 people, and the population is mainly young and middle-aged (Global Burden of Disease Study 2013 Collaborators, 2015; Olejniczak et al., 2020). As severe cases of VMC can lead to heart failure and sudden cardiac death, which seriously affect the life of patients, the timely treatment of it is very important. Current treatment for viral myocarditis remains founded on the prohibition of exercise plus supportive and symptomatic treatment, given the lack of specific antiviral therapeutic options and insufficient evidence for the use of novel immunosuppressive therapies (Tschöpe et al., 2019).

Trimetazidine, an oxidation inhibitor of free fatty acids, is often used in the treatment of heart disease with the effect of improving myocardial energy metabolism and protecting myocardium (Marzilli et al., 2019). Some studies have shown that trimetazidine has a curative effect in the treatment of VMC, which can increase the clinical effectiveness rate, improve clinical symptoms, and promote the recovery of myocardial zymogram (Yu et al., 2014). In China, traditional Chinese medicine (TCM) also has significant advantages in the treatment of VMC, showing good therapeutic effects in anti-inflammation, protecting myocardium, enhancing immune function and so on (Cao et al., 2015). Chinese herbal injection (CHI), the product of the combination of traditional Chinese medicine and modern technology, is a kind of innovative preparation with high bioavailability and good curative effect (Li et al., 2017). It is an innovative application of Chinese medicine and has been widely used in the treatment of VMC. In recent years, a large number of clinical practices have combined trimetazidine with CHI in the treatment of VMC, showing a better therapeutic effect. Liu et al (2015) conducted a meta-analysis of the efficacy and safety of *Astragalus* injection combined with trimetazidine for VMC, and the results showed that compared with conventional treatment or conventional treatment combined with trimetazidine, *Astragalus* injection combined with trimetazidine improved the clinical efficacy and reduced the cardiac zymogram level significantly. Cheng (2015) researched the efficacy of trimetazidine combined with Shenmai injection in the treatment of VMC, and the results showed that the combination could significantly shorten the improvement time of clinical symptoms and signs, and reduced B-type natriuretic peptide (BNP) and C-reactive protein (CRP) levels. Wang (2019) showed that trimetazidine combined with *Breviscapine* injection in the treatment of acute VMC is superior to trimetazidine alone in controlling the level of inflammatory cytokines, improving myocardial zymogram and relieving symptoms and so on.

Although a variety of CHIs have shown considerable efficacy in the treatment of VMC, previous studies have only explored the efficacy and safety of a single CHI combined with trimetazidine. To date, no direct or indirect comparison of different CHIs combined with trimetazidine has been reported for the treatment of VMC, meaning that it remains unclear which CHIs are most effective in the treatment of this condition. In view of this, the study aims to indirectly compare the efficacy and safety of different CHIs combined with trimetazidine in the treatment of VMC though network meta-analysis and hopes to provide some reference for clinical treatment.

## Materials and Methods

### Inclusion Criteria

All published clinical randomized controlled trials (RCTs) of CHI combined with trimetazidine in the treatment of VMC were selected. No restrictions were imposed on nationality, age, gender, and race. The control group was treated with conventional treatment or conventional treatment combined with trimetazidine. Conventional treatment included one or more of the following therapies: rest, sedation, antiarrhythmic therapy, myocardial protection, antioxidant therapy, antiviral therapy, and so on. The experimental group was treated with trimetazidine and CHI on the basis of the conventional treatment used in the control group. All the included literature should report any one of the primary or secondary outcome indicators. The primary outcome indicator was the effectiveness rate. The main reference criteria were as follows: markedly effective: clinical symptoms improved or disappeared, myocardial injury markers (myocardial zymogram or cardial troponin) returned to normal; effective: clinical symptoms relieved, myocardial injury markers improved partially, but did not fully return to normal; invalid: clinical symptoms did not improve or even further worsened, myocardial injury markers did not improve. Effectiveness rate = N (the number of markedly effective and effective cases)/N (total number of cases) × 100%. The secondary outcomes were as follows: 1) myocardial injury markers: creatine kinase (CK), creatine kinase-MB (CK-MB), lactate dehydrogenase (LDH), and cardiac troponin I (cTnI); 2) adverse reactions.

### Exclusion Criteria

1) Studies on treatment combined with other TCM-related treatment measures, such as TCM decoction, Chinese patent medicines, and acupuncture were excluded; 2) participants complicated with other diseases, such as coronary heart disease and diabetes; 3) literature works published with duplicate data; 4) the reported data were incomplete and impossible to be acquired; 5) the reported data were inconsistent with the conclusion.

### Search Strategies

To obtain RCTs of CHI combined with trimetazidine in the treatment of VMC, we searched PubMed, the Cochrane Library, Embase, China National Knowledge Infrastructure (CNKI), Wanfang database, Chinese Scientific Journals Full-Text Database (VIP), and China Biology Medicine Database (CBM) from inception to September 1, 2020. The search terms in English database were as follows: “Viral myocarditis,” “Myocarditides,” “Carditis,” “Myocarditis,” “Trimetazidine,” “Centrophène,” “Trimetazidine Dihydrochloride,” “Dihydrochloride, Trimetazidine,” “Vastarel,” “Trimétazidine Irex,” “Vasartel,” and “Idaptan.” The search terms in Chinese database included “bing du xing xin ji yan,” “qu mei ta qin,” “yan suan qu mei ta qin,” “wan shuang li,” and “san jia yang bian qin.” The search strategy of each database is shown in Supplementary Table S1.

### Literature Screening and Data Extraction

After literature retrieval, two evaluators independently conducted literature screening according to the inclusion and exclusion criteria. A preliminary screening was carried out according to the title and abstract, and then rescreening was carried out by reading the full text. Any differences in the screening results of the two evaluators were resolved by discussion with a third evaluator. After determining the included studies, the data of literature was extracted as follows: title, authors, year of publication, baseline status, methodological information, sample size, intervention measures, the course of treatment, and outcomes.

### Bias Risk Assessment

Two evaluators independently assessed the risk of bias in the included studies by using the Cochrane risk of bias tool (Higgins et al., 2011), which consisted of the following items: 1) random sequence generation; 2) allocation hiding; 3) blinding of outcome evaluators; 4) blinding of patients and trial personnel; 5) incomplete result data; 6) selective reporting; and 7) other biases. The risk assessment criteria are divided into low, high, and uncertain bias risk. Any differences in the assessment results of the two evaluators were resolved by discussion with a third evaluator. The bias risk assessment results of the included studies were visualized by RevMan5.3 software.

### Statistical Analysis

All statistical analysis was performed using Stata 16.0 software (Stata Company). For dichotomous variables (effectiveness rate and adverse reaction rate), odds ratios (OR), and 95% confidence intervals (CI) were used to assess effect size, whereas the effect size of continuous variables (myocardial injury markers) was assessed using the standard mean difference (SMD) and 95% CI. Considering the expected heterogeneity in the included studies, a random-effects model was used for statistical analysis. If the data could not be meta-analyzed, we would conduct a descriptive analysis. Based on the classical frequency, the random-effects model was selected under the “Network” command in the analysis software to perform the network meta-analysis. A network diagram of interventions was constructed to show the relationships between interventions. Where no closed loop was formed, the consistency model was used for analysis. When a closed loop was observed, an overall inconsistency check was performed. If *p* value > 0.05, it indicated that the overall inconsistency was not significant, otherwise, it indicated that the overall inconsistency was significant. At the same time, the inconsistency test of the loop was carried out to determine inconsistency between direct and indirect evidence by calculating the absolute difference between these types of evidence, expressed as an inconsistency factor (IF). Whereas 95% CI of the IF values contained 0, and the inconsistency of direct and indirect evidence was considered not significant. Otherwise, the inconsistency was deemed significant. When no inconsistency was apparent, the consistency model was used for analysis. Otherwise, the inconsistency model was selected for analysis. The value of surface under the cumulative ranking (SUCRA) was subsequently calculated and the number of iterations set at 5,000. Intervention measures were sorted based on SUCRA value. The larger the SUCRA value, the higher the ranking, indicating that the intervention was more likely to be the best intervention. Funnel plots were used to evaluate publication bias in the included studies.

## Results

### Literature Search and Screening

**FIGURE 1:**
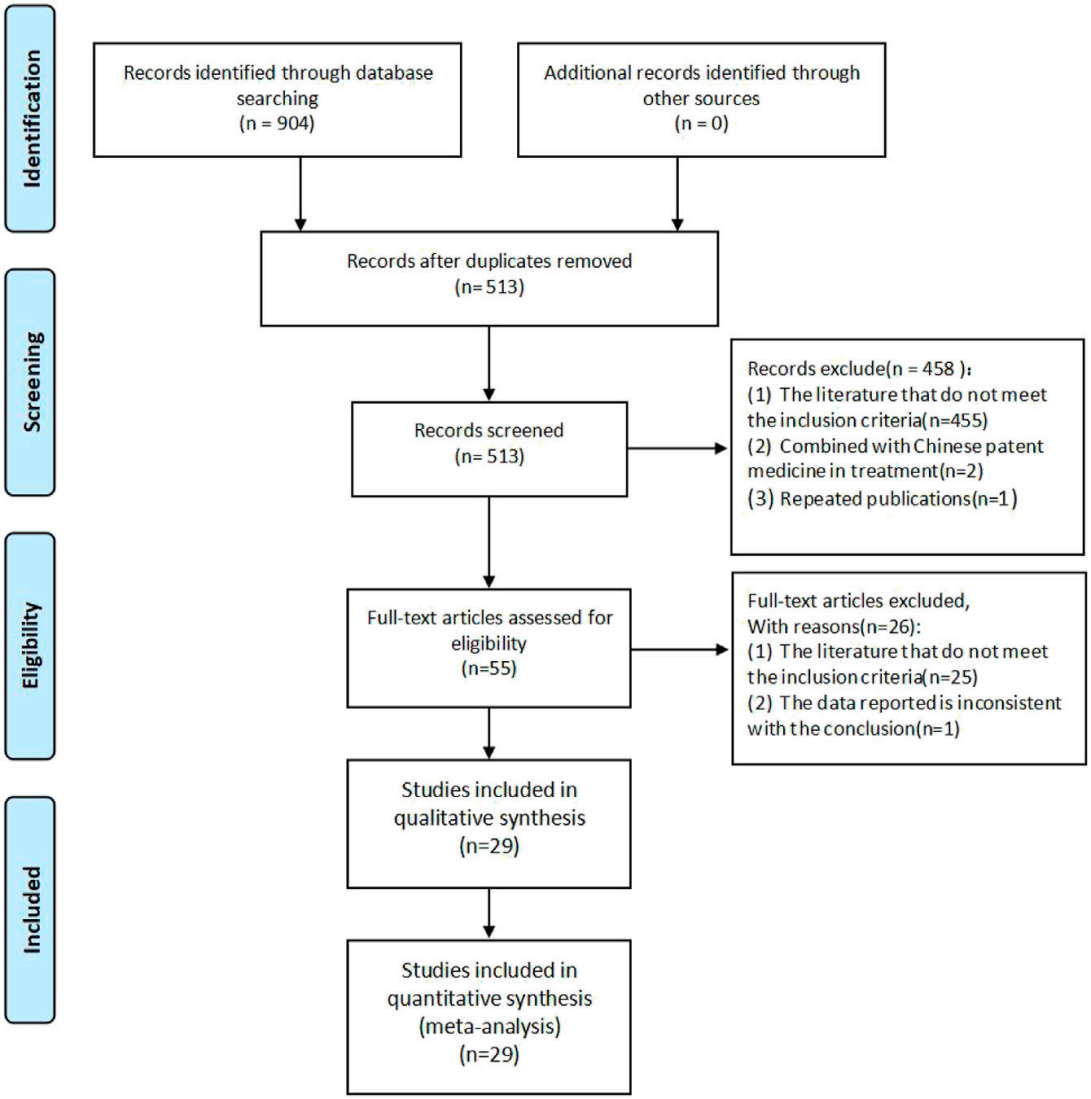
Flow diagram of literature screening process.

A total of 904 literature works were obtained. Among them, there were 3 from PubMed, 7 from the Cochrane Library, 4 from Embase, 453 from CNKI, 172 from Wanfang database, 135 from VIP, and 130 from CBM. After screening, a total of 29 studies were included. The literature screening process is shown in Figure 1.

### Basic Characteristics of Included Studies

**FIGURE 2:**
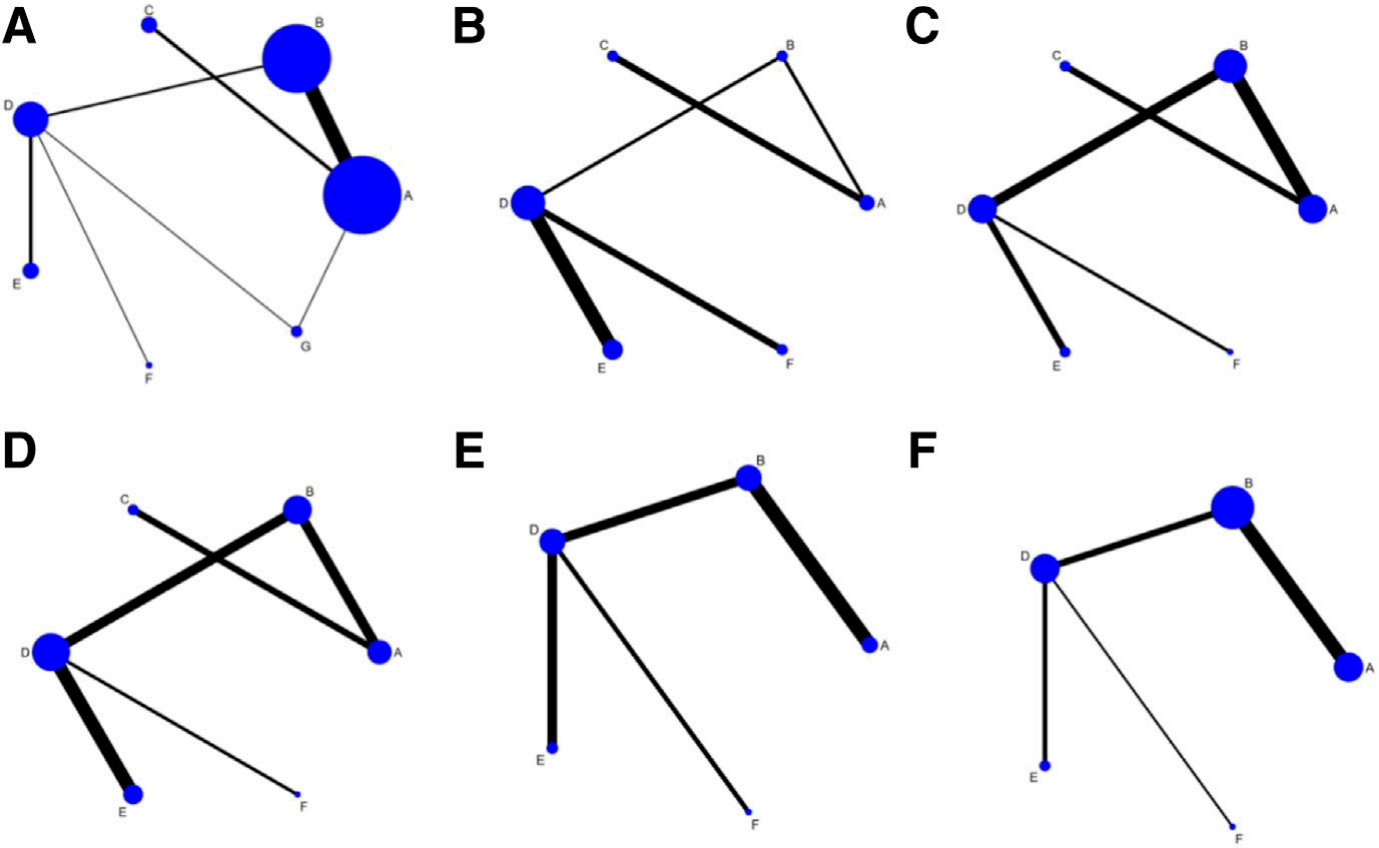
Network diagrams of outcome indicators.**(A)** effectiveness rate; **(B)** CK; **(C)** CK-MB; **(D)** LDH; **(E)** cTnI; **(F)** adverse reaction rate. A: conventional treatment; B: conventional treatment + trimetazidine + *Astragalus* injection; C: conventional treatment + trimetazidine + Shenfu injection; D: conventional treatment + trimetazidine; E: conventional treatment + trimetazidine + S*alviae miltiorrhizae* and *ligustrazine hydrochloride* injection; F: conventional treatment + trimetazidine + *Breviscapine* injection; G: conventional treatment + trimetazidine + Shenmai injection.

29 studies enrolled a total of 2,687 patients. All the included studies were conducted in China, and the literature was published from 2009 to 2020. The baselines of all studies were comparable between the experimental and control groups. In terms of intervention measures, the control groups of 18 studies were treated with conventional treatment alone, whereas the experimental groups were treated with CHI and trimetazidine on the basis of the control groups. Among them, 14 studies were combined with *Astragalus* injection, 3 studies combined with Shenfu injection, and 1 study combined with Shenmai injection. In the other 11 studies, the control groups were treated with conventional treatment combined with trimetazidine, whereas the experimental groups were treated with CHI on the basis of the control group. Among them, 4 studies combined with *Astragalus* injection, 4 studies combined with *Salviae miltiorrhizae* and *ligustrazine hydrochloride* injection, 2 studies combined with *Breviscapine* injection, and 1 study combined with Shenmai injection. The detailed information of CHIs used in included studies and the chemical analysis of them are shown in Supplementary Tables S2, S3. In the course of treatment, the shortest was 2 weeks and the longest was 8 weeks. In the outcome indicators, 25 studies reported the effectiveness rate, 10 studies reported the level of CK, 12 studies reported the level of CK-MB, 13 studies reported the level of LDH, 8 studies reported the level of cTnI, and 20 studies reported the adverse reactions during treatment. The basic characteristics of included studies are detailed in Table 1. For the convenience of description, A refers to conventional treatment, B refers to conventional treatment combined with trimetazidine and *Astragalus* injection, C refers to conventional treatment combined with trimetazidine and Shenfu injection, D refers to conventional treatment combined with trimetazidine, E refers to conventional treatment combined with trimetazidine and *Salviae miltiorrhizae* and *ligustrazine hydrochloride* injection, F refers to conventional treatment combined with trimetazidine and *Breviscapine* injection, and G refers to conventional treatment combined with trimetazidine and Shenmai injection. The network diagrams of the seven interventions in different outcome indicators are shown in Figure 2.

**TABLE 1:** Basic characteristics of the included studies.

Study	Age (mean or range)	Sample size	Intervention		Courses	Outcome
	C/E	C/E	C	E		
Chen (2011)	17–46/17–46	32/32	Conventional treatment	Conventional treatment + trimetazidine 20 mg/d + *Astragalus* injection 40 ml/d	*Astragalus* injection 2 w	(1) (4) (6)
					Others 8 w	
Chen (2014)	57.8/57.8	80/80	Conventional treatment	Conventional treatment + trimetazidine 20 mg/d + *Astragalus* injection 50 ml/d	1 m	(1) (6)
Dai (2018)	25.4 ± 8.6/25.6 ± 7.9	20/20	Conventional treatment	Conventional treatment + trimetazidine 60 mg/d + *Astragalus* injection 50 ml/d	1 m	(1) (2) (3) (4) (6)
Ge,et al. (2010)	27.2 ± 11.2/26.8 ± 10.7	30/30	Conventional treatment	Conventional treatment + trimetazidine 20 mg/d + *Astragalus* injection 40 ml/d	*Astragalus* injection 2w	(1) (6)
					Others 8w	
Ma (2012)	22.48 ± 7.2/23.56 ± 8.5	46/62	Conventional treatment	Conventional treatment + trimetazidine 60 mg/d + *Astragalus* injection 20 g/d	2w	(1) (3) (5) (6)
Pu (2013)	—	67/79	Conventional treatment	Conventional treatment + trimetazidine 60 mg/d + *Astragalus* injection 50 ml/d	4w	(1) (6)
Shao,et al. (2012)	27 ± 11/28 ± 7	48/50	Conventional treatment	Conventional treatment + trimetazidine 60 mg/d + *Astragalus* injection 10–20 ml/d	*Astragalus* injection 2w	(1) (6)
					Others 8w	
Sun (2013)	30 ± 5/28 ± 5	40/40	Conventional treatment	Conventional treatment + trimetazidine 60 mg/d + *Astragalus* injection 20 g/d	4w	(1) (6)
Wang (2016)	55.6 ± 2.4/56.3 ± 2.8	37/37	Conventional treatment	Conventional treatment + trimetazidine 60 mg/d + *Astragalus* injection 20 ml/d	2w	(1) (3) (5) (6)
Wang (2010)	31 ± 10/30 ± 10	50/50	Conventional treatment	Conventional treatment + trimetazidine 60 mg/d + *Astragalus* injection 30 ml/d	3w	(1) (6)
Xu and Zhang (2011)	14–40/13–41	30/60	Conventional treatment	Conventional treatment + trimetazidine 60 mg/d + *Astragalus* injection 40 ml/d	2w	(1) (4)
Yang (2009)	31 ± 10/32 ± 10	43/45	Conventional treatment	Conventional treatment + Trimetazidine 60 mg/d + *Astragalus* injection 50 ml/d	4w	(1) (6)
Zhang,et al. (2015)	25.2 ± 8.5/26.2 ± 8.5	30/30	Conventional treatment	Conventional treatment + trimetazidine 60 mg/d + *Astragalus* injection 30 ml/d	—	(1)
Yu (2014)	32.4 ± 5.6/32.9 ± 6.1	51/51	Conventional treatment	Conventional treatment + trimetazidine 60 mg/d + *Astragalus* injection 50 ml/d	4w	(1) (3) (5) (6)
Zhang,et al. (2016)	44.1 ± 7.2/44.5 ± 7.8	40/40	Conventional treatment	Conventional treatment + trimetazidine 60 mg/d + Shenfu injection 50 ml/d	4w	(1) (2) (3) (4)
Sun and Sun (2018)	53.1 ± 5.8/52.4 ± 5.3	51/51	Conventional treatment	Conventional treatment + trimetazidine 60 mg/d + Shenfu injection 50 ml/d	Shenfu injection 2w	(1)
					Others 4w	
Gao (2019)	46.21 ± 2.57/46.73 ± 2.10	39/39	Conventional treatment	Conventional treatment + trimetazidine 60 mg/d + Shenfu injection 40–200 ml/d	—	(1) (2) (3) (4)
Pang and Huang (2013)	10 m–12/10 m–12	33/33	Conventional treatment	Conventional treatment + trimetazidine 0.3–0.5 mg/kg/d + Shenmai injection 0.5–1 ml/kg/d	2w	(1)
Wang (2012)	60 ± 9/55 ± 11	35/35	Conventional treatment + trimetazidine 60 mg/d	Conventional treatment + trimetazidine 60 mg/d + *Astragalus* injection 50 ml/d	4w	(1) (6)
Zheng (2019)	49.15 ± 16.18/48.47 ± 15.25	44/45	Conventional treatment + trimetazidine 60 mg/d	Conventional treatment + trimetazidine 60 mg/d + *Astragalus* injection 10–20 ml/d	6w	(3) (4) (6)
Cui (2018)	55.2 ± 6.5/54.8 ± 6.3	35/35	Conventional treatment + trimetazidine 60 mg/d	Conventional treatment + trimetazidine 60 mg/d + *Astragalus* injection 40 ml/d	4w	(2) (3) (4) (5)
Wei,et al. (2020)	70.3 ± 7.8/70.9 ± 7.5	90/90	Conventional treatment + trimetazidine 60 mg/d	Conventional treatment + trimetazidine 60 mg/d + *Astragalus* injection 20 ml/d	2w	(1) (3) (4) (5) (6)
Zhu,et al. (2020)	5.81 ± 1.22/5.7 ± 1.15	46/46	Conventional treatment + trimetazidine 20 mg/d	Conventional treatment + trimetazidine 20 mg/d + *Salviae miltiorrhizae* and *ligustrazine hydrochloride* injection 5 ml/d	30 d	(1) (2) (3) (4) (5) (6)
Wang (2020)	9.91 ± 1.27/9.89 ± 1.31	50/50	Conventional treatment + trimetazidine 60 mg/d	Conventional treatment + trimetazidine 60 mg/d + *Salviae miltiorrhizae* and *ligustrazine hydrochloride* injection 5 ml/d	30 d	(2) (3) (4) (5)
Chen and Zeng (2018)	41.2 ± 6.8/41.8 ± 6.9	49/49	Conventional treatment + trimetazidine 60 mg/d	Conventional treatment + trimetazidine 60 mg/d + *Salviae miltiorrhizae* and *ligustrazine hydrochloride* injection 5 ml/d	1 m	(1) (2) (4)
Li and Gao (2016)	43.9 ± 7.9/43.3 ± 7.5	55/55	Conventional treatment + trimetazidine 20 mg/d	Conventional treatment + trimetazidine 20 mg/d + *Salviae miltiorrhizae* and *ligustrazine hydrochloride* injection 5 ml/d	30 d	(1) (2) (4) (6)
Miao (2019)	51.17 ± 2.67/50.64 ± 3.2	49/49	Conventional treatment + trimetazidine 60 mg/d	Conventional treatment + trimetazidine 60 mg/d + *Breviscapine* injection 5 ml/d	1 m	(2) (6)
Wang (2017)	42.1 ± 15.9/43.2 ± 15.6	62/62	Conventional treatment + trimetazidine 60 mg/d	Conventional treatment + trimetazidine 60 mg/d + *Breviscapine* injection 5 ml/d	4 w	(1) (2) (3) (4) (5) (6)
He (2014)	33.52 ± 1.14/33.52 ± 1.14	30/30	Conventional treatment + trimetazidine 60 mg/d	Conventional treatment + trimetazidine 60 mg/d + Shenmai injection 2–4 ml/d	4 w	(1) (6)

C: control group; E: experimental group; d: day; w: week; m: month; (1): effectiveness rate; (2): CK; (3): CK-MB; (4): LDH; (5): cTnI; (6): adverse reactions.

### Bias Risk Assessment of Included Studies

**FIGURE 3:**
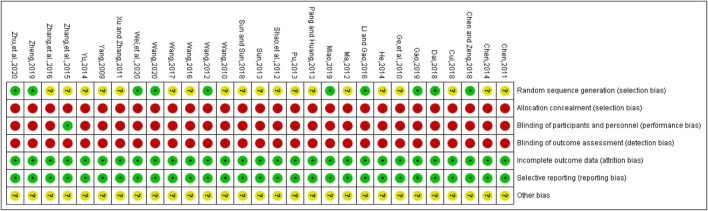
Risk of bias for each included study. Red represents high risk of bias; green represents low risk of bias; yellow represents uncertain risk of bias.

The methodological quality of the included studies was generally low. In the generation of random sequences, 9 studies used random number tables, and 1 study used lottery randomization, so they were at low bias risk. Other studies only mentioned randomness and did not describe random methods, so they were at uncertain bias risk. None studies adopted random allocation hiding and blinding to outcome evaluators, which belonged to high risk bias. One study applied blinding to patients and trial personnel, which belonged to low bias risk, while other studies did not adopt blinding, and they all belonged to high bias risk. All the data were reported completely, and there were no evidences to support the selective reporting of outcomes, so it belonged to low bias risk. Whether there were other biases in all included studies could not be judged clearly, so they were at uncertain bias risk. The bias risk assessment results of included studies are detailed in Figure 3.

### Outcome Indicators

#### Effectiveness Rate

**FIGURE 4:**
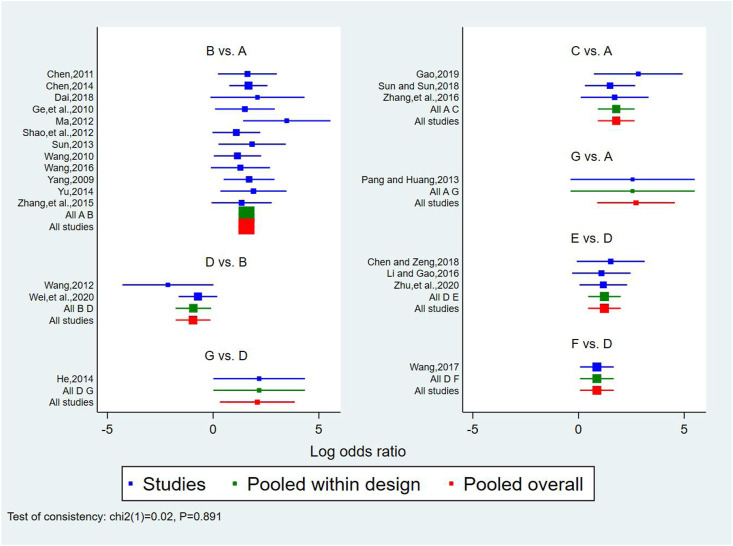
Forest diagram of direct comparison of effectiveness rate. A: conventional treatment; B: conventional treatment + trimetazidine + *Astragalus* injection; C: conventional treatment + trimetazidine + Shenfu injection; D: conventional treatment + trimetazidine; E: conventional treatment + trimetazidine + *Salviae miltiorrhizae* and *ligustrazine hydrochloride* injection; F: conventional treatment + trimetazidine + *Breviscapine* injection; G: conventional treatment + trimetazidine + Shenmai injection.

**FIGURE 5:**
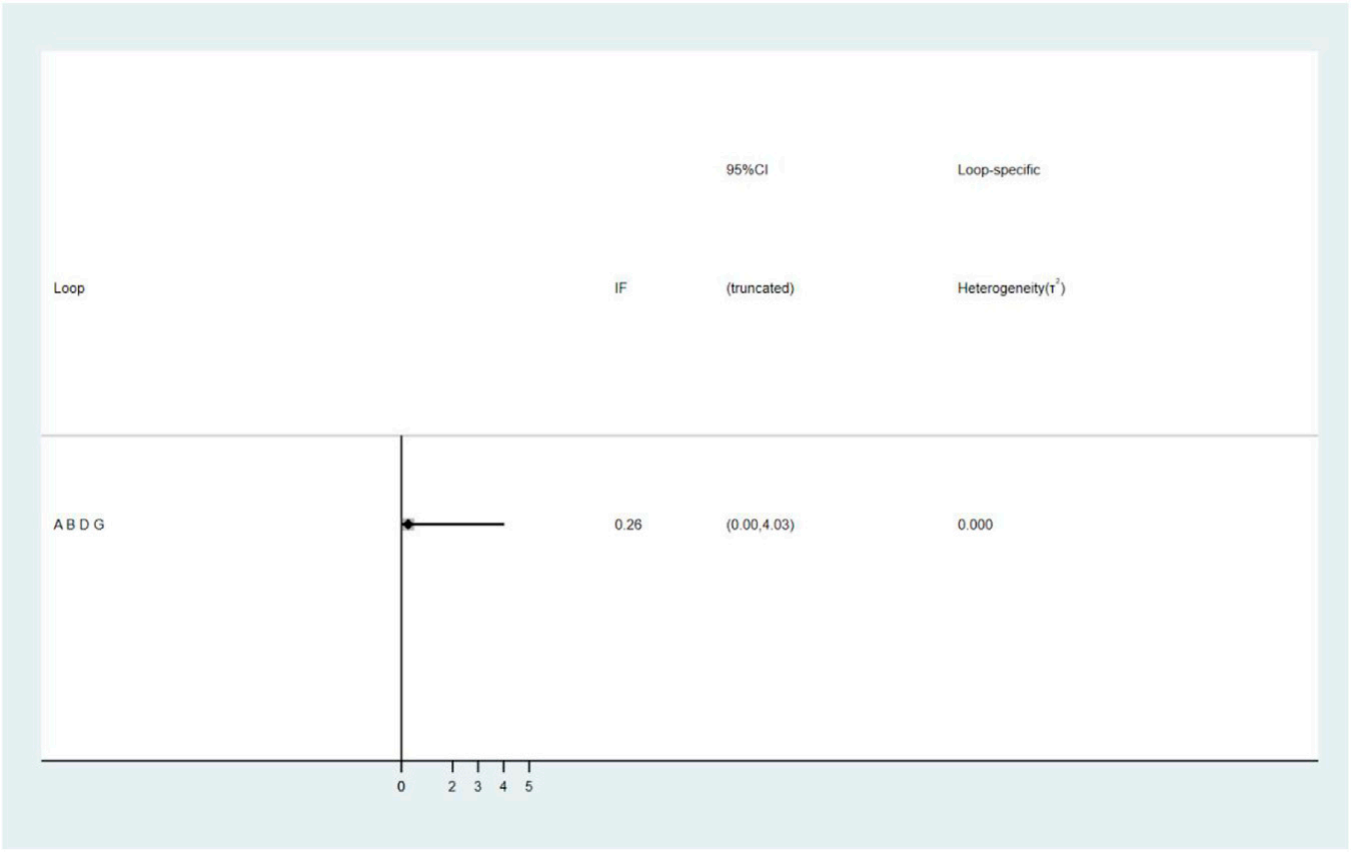
The result of loop inconsistency test. A: conventional treatment; B: conventional treatment + trimetazidine + *Astragalus* injection; D: conventional treatment + trimetazidine; G: conventional treatment + trimetazidine + Shenmai injection.

A total of 25 studies reported effectiveness rate, of which 2 studies (Xu and Zhang, 2011; Pu, 2013) had data distortion and were not combined in the analysis. The remaining 23 studies constituted 7 pairs of direct comparisons, which involved 5 types of CHIs. Four types of intervention measures (A and G) formed a quadrilateral closed loop, as shown in Figure 2A. The overall inconsistency test result had a *p* value = 0.891, indicating that the inconsistency was not significant, as shown in Figure 4. The inconsistency test of the loop resulted in an IF = 0.26 and 95% CI (0.00, 4.03), indicating that the inconsistency between the direct and indirect evidence was not significant, as shown in Figure 5. The consistent model was therefore adopted for network meta-analysis.

A total of 21 pairs were compared among the 7 intervention measures, 9 of which showed statistically significant differences (*p* < 0.05). On the basis of conventional treatment, combining with *Astragalus* injection and trimetazidine [OR = 4.83, 95% CI (3.30, 7.09)], or Shenfu injection and trimetazidine [OR = 5.96, 95% CI (2.50, 14.23)], or *Salviae miltiorrhizae* and *ligustrazine hydrochloride* injection and trimetazidine [OR = 6.38, 95% CI (1.96, 20.77)], or *Breviscapine* injection and trimetazidine [OR = 4.46, 95% CI (1.34, 14.80)], or Shenmai injection and trimetazidine [OR = 15.17, 95% CI (2.41, 95.25)] was superior to conventional treatment alone with respect to effectiveness rate. In addition to the Shenfu injection, on the basis of conventional treatment plus trimetazidine, combining with *Astragalus* injection [OR = 2.58, 95% CI(1.14, 5.86)], or *Salviae miltiorrhizae* and *ligustrazine hydrochloride* injection [OR = 3.40, 95% CI(1.58, 7.34)] or *Breviscapine* injection [OR = 2.38, 95% CI (1.07, 5.28)], or Shenmai injection [(OR = 8.09, 95% CI (1.38, 47.59)] was superior to conventional treatment plus trimetazidine in terms of effectiveness rate. The comparisons among the 5 types of injections and between conventional treatment and conventional treatment plus trimetazidine showed no statistically significant difference (*p* > 0.05). The above results are detailed in Table 2.

**TABLE 2:** The network meta-analysis results of the outcome indicators.

Intervention	Outcome indicators (OR/SMD, 95% CI)					
	Effectiveness rate	CK	CK-MB	LDH	cTnI	Adverse reaction rate
G vs. F	3.40, (0.49, 23.72)	—	—	—	—	—
G vs. E	2.38, (0.34, 16.40)	—	—	—	—	—
G vs. D	**8.09, (1.38, 47.59)**	—	—	—	—	—
G vs. C	2.54, (0.33, 19.44)	—	—	—	—	—
G vs. B	3.14, (0.50, 19.52)	—	—	—	—	—
G vs. A	**15.17, (2.41, 95.25)**	—	—	—	—	—
F vs. D	**2.38, (1.07, 5.28)**	**−1.90, (−3.07, −0.72)**	−1.68, (−4.24, 0.87)	−0.97, (−3.86, 1.92)	−3.33, (−57.22, 50.56)	0.23, (0.03, 2.18)
F vs. C	0.75, (0.17, 3.29)	−1.15, (−4.17, 1.88)	−1.54, (−5.26.2.19)	−0.93, (−5.21, 3.35)	—	—
F vs. B	0.92, (0.29, 2.89)	0.21, (−1.87, 2.28)	1.04, (−1.93.4.01)	0.36, (−2.98, 3.70)	−0.48, (−66.28, 65.33)	0.19, (0.02, 2.14)
F vs. A	**4.46, (1.34, 14.80)**	−2.73, (−5.51, 0.05)	−2.45, (−5.69.0.80)	−1.89, (−5.64, 1.87)	−31.67, (−104.23, 40.89)	0.19, (0.02, 2.34)
E vs. F	1.43, (0.47, 4.32)	−0.17, (−1.61, 1.28)	−0.62, (−3.77.2.52)	**−3.52, (−6.77, −0.27)**	1.08, (−64.92, 67.08)	6.57, (0.55, 78.19)
E vs. D	**3.40, (1.58, 7.34)**	**−2.06, (−2.90, −1.22)**	**−2.31, (−4.13, −0.48)**	**−4.49, (−5.97, −3.00)**	−2.25, (−40.36, 35.86)	1.54 (0.52.4.53)
E vs. C	1.07, (0.25, 4.64)	−1.31, (−4.22, 1.60)	−2.16, (−5.42.1.10)	**−4.45, (−7.94, −0.96)**	—	—
E vs. B	1.32, (0.43, 4.06)	0.04, (−1.87, 1.95)	0.42, (−1.95.2.78)	**−3.16, (−5.40, −0.92)**	0.61, (−53.04, 54.25)	1.28, (0.32.5.15)
E vs. A	**6.38, (1.96, 20.77)**	**−2.90, (−5.55, −0.24)**	**−3.07, (−5.78, −0.36)**	**−5.40, (−8.23, −2.58)**	−30.59, (−92.34, 31.16)	1.27, (0.27.6.02)
C vs. D	3.18, (0.91, 11.10)	−0.75, (−3.54, 2.04)	−0.15, (−2.85.2.56)	−0.04, (−3.20, 3.12)	—	—
C vs. A	**5.96, (2.50, 14.23)**	**−1.59, (−2.78, −0.40)**	−0.91, (−2.73.0.91)	−0.95, (−3.01, 1.10)	—	—
B vs. C	0.81, (0.31, 2.10)	−1.35, (−3.55, 0.85)	**−2.58, (−0.33, −4.82)**	−1.29, (−3.98, 1.39)	—	—
B vs. D	**2.58, (1.14, 5.86)**	**−2.10, (−3.81, −0.39)**	**−2.73, (−4.23, −1.22)**	−1.33, (−3.01, 0.34)	−2.86, (−40.76, 35.05)	1.21, (0.50.2.91)
B vs. A	**4.83, (3.30, 7.09)**	**−2.94, (−4.79, −1.09)**	**−3.49, (−4.80, −2.17)**	**−2.25, (−3.97, −0.53)**	−31.19, (−62.36, −0.03)	0.99, (0.50.1.98)
A vs. D	0.53, (0.22, 1.31)	0.84, (−1.68, 3.36)	0.76, (−1.24.2.76)	0.92, (−1.49, 3.32)	28.34, (−20.43, 77.11)	1.21, (0.40.3.72)

OR, odds ratio; SMD, standardized mean difference; CI, confidence interval; A, conventional treatment; B, conventional treatment + trimetazidine + Astragalus injection; C, conventional treatment + trimetazidine + Shenfu injection; D, conventional treatment + trimetazidine; E, conventional treatment + trimetazidine + Salviae miltiorrhizae and ligustrazine hydrochloride injection; F, conventional treatment + trimetazidine + Breviscapine injection; G, conventional treatment + trimetazidine + Shenmai injection.

**FIGURE 6:**
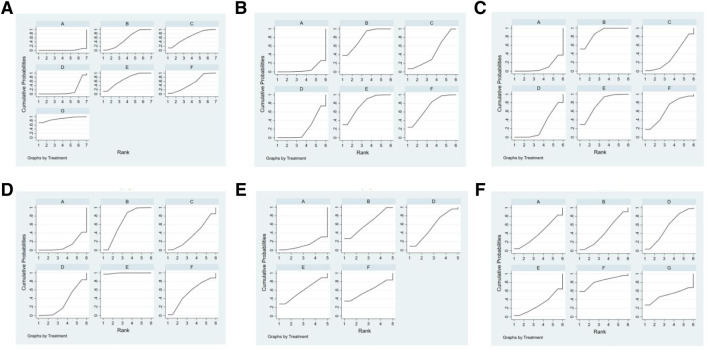
SUCRA of outcome indicators.**(A)**:effectiveness rate; **(B)**:CK; **(C)**: CK-MB; **(D)**: LDH; **(E)**: cTnI; **(F)**: adverse reaction rate. A: conventional treatment; B: conventional treatment + trimetazidine + *Astragalus* injection; C: conventional treatment + trimetazidine + Shenfu injection; D: conventional treatment + trimetazidine; E: conventional treatment + trimetazidine + *Salviae miltiorrhizae* and *ligustrazine hydrochloride* injection; F: conventional treatment + trimetazidine + *Breviscapine* injection; G: conventional + treatment + trimetazidine + Shenmai injection.

Ranking the 5 types of injections according to the SUCRA resulted in the following hierarchy: Shenmai injection (89.2%), *Salviae miltiorrhizae* and *ligustrazine hydrochloride* injection (69.1%), Shenfu injection (65.5%), *Astragalus* injection (55.2%), and *Breviscapine* injection (52.3%), as shown in Figure 6A.

#### Creatine Kinase

A total of 10 studies reported the level of CK, which constituted 5 pairs of direct comparisons, involved 4 types of CHIs and 6 types of intervention measures. The network diagram formed is shown in Figure 2B. Since it did not form a closed loop, no inconsistency test was carried out.

A total of 15 pairs were compared among the 6 intervention measures, 6 of which showed statistically significant differences (*p* > 0.05). On the basis of conventional treatment, combining with *Astragalus* injection and trimetazidine [SMD = −2.94, 95% CI (−4.79,−1.09)], or Shenfu injection and trimetazidine [SMD = −1.59, 95% CI (−2.78, −0.40)], or *Salviae miltiorrhizae* and *ligustrazine hydrochloride* injection and *trimetazidine* [SMD = −2.90, 95% CI (−5.55,−0.24)] was superior to conventional treatment alone on reducing the level of CK. On the basis of conventional treatment plus trimetazidine, combining with *Astragalus* injection [SMD = −2.10, 95% CI (−3.81, −0.39)], or *Salviae miltiorrhizae* and *ligustrazine hydrochloride* injection [SMD = −2.06, 95% CI (−2.90, −1.22)] or *Breviscapine* injection [SMD = −1.90, 95% CI (−3.07, −0.72)] was superior to conventional treatment plus trimetazidine on reducing the level of CK. The comparisons among the 4 types of injections and between conventional treatment and conventional treatment plus trimetazidine showed no statistically significant difference (*p* > 0.05). The above results are detailed in Table 2.

Ranking the 4 injections based on the SUCRA value, the results were as follows: *Astragalus* injection (79.5%), *Salviae miltiorrhizae* and *ligustrazine hydrochloride* injection (77.1%), *Breviscapine* injection (71.7%), and Shenfu injection (44.5%), as shown in Figure 6B.

#### Creatine Kinase-MB

A total of 12 studies reported the level of CK-MB, which constituted 5 pairs of direct comparisons, involved 4 types of CHIs and 6 types of intervention measures. The network diagram formed is shown in Figure 2C. It did not form a closed loop, so there was no inconsistency test.

A total of 15 pairs of comparisons were formed among the 6 intervention measures, 5 of which were statistically significant (*p* < 0.05). On the basis of conventional treatment, combining with *Astragalus* injection and trimetazidine [SMD = −3.49, 95% CI (−4.80,−2.17)], or *Salviae miltiorrhizae* and *ligustrazine hydrochloride* injection and trimetazidine [SMD = −3.07, 95% CI (−5.78,−0.36)] was superior to conventional treatment alone on reducing the level of CK-MB. On the basis of conventional treatment plus trimetazidine, combining with *Astragalus* injection [SMD = −2.73, 95% CI (−4.23,−1.22)], or *Salviae miltiorrhizae* and *ligustrazine hydrochloride* injection [SMD = −2.31, 95% CI (−4.13, −0.48)] was superior to conventional treatment plus trimetazidine on reducing the level of CK-MB. The comparisons among the 4 types of injections showed that on the basis of conventional treatment plus trimetazidine, combining with *Astragalus* injection was superior to Shenfu injection [SMD = −2.58, 95% CI (−0.33, −4.82)] on reducing the level of CK-MB. The other 10 pair comparisons showed no statistically significant difference (*p* > 0.05). The above results are detailed in Table 2.

Based on the SUCRA value, the 4 types of injections were ranked as follows: *Astragalus* injection (87.5%), *Salviae miltiorrhizae* and *ligustrazine hydrochloride* injection (77.9%), *Breviscapine* injection (64.3%), and Shenfu injection (33.9%), as shown in Figure 6C.

#### Lactate Dehydrogenase

A total of 13 studies reported the level of LDH, which constituted 5 pairs of direct comparisons, involved 4 types of CHIs and 6 types of intervention measures. The network diagram formed is shown in Figure 2D. No closed loop was constituted and no inconsistency test was performed.

A total of 15 pairs of comparisons were formed among the 6 intervention measures, 6 of which were statistically significant (*p* < 0.05). On the basis of conventional treatment, combining with *Astragalus* injection and trimetazidine [SMD = −2.25, 95% CI (−3.97, −0.53)], or *Salviae miltiorrhizae* and *ligustrazine hydrochloride* injection and trimetazidine [SMD = −5.40, 95% CI (−8.23, −2.58)] was superior to conventional treatment alone on reducing the level of LDH. On the basis of conventional treatment plus trimetazidine, combining with *Salviae miltiorrhizae* and *ligustrazine hydrochloride* injection [SMD = −4.49, 95% CI (−5.97,−3.00)]was superior to conventional treatment plus trimetazidine on reducing the level of LDH. The comparisons among the 4 types of injections showed that on the basis of conventional treatment plus trimetazidine, combining with *Salviae miltiorrhizae* and *ligustrazine hydrochloride* injection was superior to *Astragalus* injection [SMD = −3.16, 95% CI (−5.40, −0.92)] or Shenfu injection [SMD = −4.45, 95% CI (−7.94, −0.96)] or *Breviscapine* injection [SMD = −3.52, 95% CI (−6.77, −0.27)] on reducing LDH. The other 9 pairs comparisons were not statistically significant (*p* > 0.05). The above results are detailed in Table 2.

Ranking the 4 injections based on the SUCRA value, the results were as follows: *Salviae miltiorrhizae* and *ligustrazine hydrochloride* injection (99.4%), *Astragalus* injection (66.7%), *Breviscapine* injection (54.3%), and Shenfu injection (36.4%), as shown in Figure 6D.

#### Cardiac Troponin I

A total of 8 studies reported the level of cTnI, which constituted 4 pairs of direct comparisons, involved 3 types of CHIs and 5 types of intervention measures. The network diagram formed is shown in Figure 2E. Since it did not constitute a closed loop, the inconsistency test was not performed.

The results showed that there were no statistically significant differences among 10 pairs of comparisons of the 5 intervention measures (*p* > 0.05). The above results are detailed in Table 2.

Based on the SUCRA value, the 3 injections were ranked as follows: *Astragalus* injection (63.2%), *Salviae miltiorrhizae* and *ligustrazine hydrochloride* injection (59.4%), and *Breviscapine* injection (59.4%), as shown in Figure 6E.

#### Adverse Reaction Rate

Eight studies reported no adverse reactions during treatment, and 12 studies reported the number of cases of adverse reactions. The adverse reactions mainly include dizziness, headache, abdominal discomfort, nausea and vomiting, poor appetite, diarrhea, and rash. In terms of the adverse reaction rate, 12 studies consisted 4 pairs of comparisons, involved 3 types of CHIs and 5 types of intervention measures. The network diagram formed is shown in Figure 2F. Since it did not constitute a closed loop, the inconsistency test was not performed. The results showed that there were no statistically significant differences among 10 pairs comparisons of the 5 intervention measures (p > 0.05). The above results are detailed in Table 2.

Ranking the three injections based on the SUCRA value, the results were as follows: *Breviscapine* injection (90.4%), *Astragalus* injection (39.3%), and *Salviae miltiorrhizae* and *ligustrazine hydrochloride* injection (26.1%), as shown in Figure 6F.

#### Assessment of Publication Bias

**FIGURE 7:**
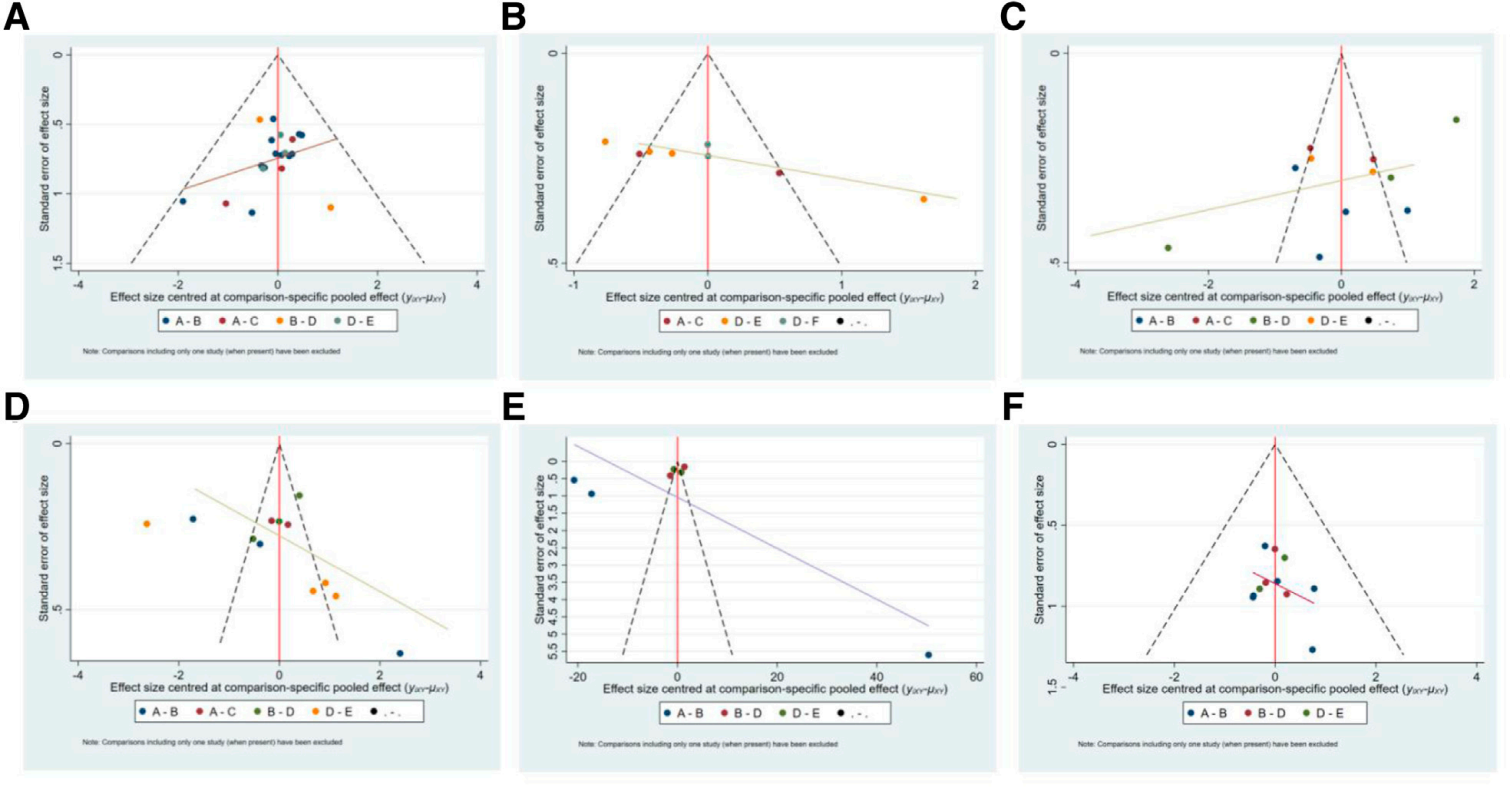
The funnel plot of outcome indicators. **A**:effectiveness rate; **B**:CK; **C**:CK-MB; **D**:LDH; **E**:cTnI; **F**:adverse reaction rate. A: conventional treatment; B: conventional treatment + trimetazidine + *Astragalus* injection; C: conventional treatment + trimetazidine + Shenfu injection; D: conventional treatment + trimetazidine; E: conventional treatment + trimetazidine + *Salviae miltiorrhizae* and *ligustrazine hydrochloride* injection; F: conventional treatment + trimetazidine + *Beviscapine* injection.

The funnel plots have poor symmetry, indicating that there was some publication bias in the included studies, which may be caused by small sample effects, as shown in Figure 7.

## Discussion

In the present study, we conducted a search of the RCTs of CHIs combined with trimetazidine in the treatment of VMC, and adopted a network meta-analysis approach to evaluate the clinical efficacy and safety of different CHIs combined with trimetazidine. A total of 29 RCTs were included in this analysis, representing 7 types of intervention measures and 5 types of CHIs. Our findings indicate that, in terms of effectiveness rate and reduction of the CK and CK-MB levels, *Astragalus* injection or *Salviae miltiorrhizae* and *ligustrazine hydrochloride* injection combined with trimetazidine was superior to conventional treatment alone or conventional treatment combined with trimetazidine. Moreover, *Salviae miltiorrhizae* and *ligustrazine hydrochloride* injection combined with trimetazidine was also superior to conventional treatment or conventional treatment combined with trimetazidine in reducing the level of LDH, and *Astragalus* injection combined with trimetazidine was also superior to conventional treatment in reducing the level of LDH. Shenmai injection or *Breviscapine* injection combined with trimetazidine was better than conventional treatment or conventional treatment combined with trimetazidine in terms of effectiveness rate. *Breviscapine* injection combined with trimetazidine was superior to conventional treatment combined with trimetazidine in reducing the level of CK. Shenfu injection combined with trimetazidine was superior to conventional treatment alone in terms of effectiveness rate and reduction of CK level. The comparison of different CHIs showed that *Astragalus* injection was superior to Shenfu injection in reducing the level of CK-MB. In reducing the level of LDH, *Salviae miltiorrhizae* and *ligustrazine hydrochloride* injection was superior to *Astragalus* injection, Shenfu injection, and *Breviscapine* injection. In terms of safety, there was no significant difference among different interventions. Based on the ranking of the SUCRA value, Shenmai injection may be superior to other injections in terms of effectiveness rate, *Astragalus* injection and *Salviae miltiorrhizae* and *ligustrazine hydrochloride* injection may be superior to other injections in reducing myocardial injury markers. However, different CHIs have different properties. Some tend to replenish qi, some tend to activate blood circulation, some tend to tonify yang, and some tend to nourish yin. Therefore, for the selection of CHI, it is better to choose based on the performance of patients and syndrome differentiation of traditional Chinese medicine in clinical practice, which may be able to harvest a better clinical efficacy.

As a myocardial metabolic agent, trimetazidine can optimize the oxidation of fatty acids and glucose, improve myocardial metabolism, inhibit cardiomyocyte apoptosis, reduce cardiac remodeling, improve cardiac function and so on, which is often used in the treatment of cardiovascular diseases (Li et al., 2020). *Astragalus* injection is extracted from the root of *Astragalus mongholicus Bunge*. Previous studies have shown that the application of *Astragali Radix* can improve the survival rate and relieve the pathological changes of mice with VMC induced by coxsackievirus B3 (Chen et al., 2006). Astragaloside IV plays a cardioprotective role in experimental animals with VMC through a variety of signal pathways, such as antimyocardial remodeling, antivirus, anti-oxidation, anti-inflammation, anti-apoptosis, and reducing myocardial fibrosis (Zhuang et al., 2019). *Salviae miltiorrhizae* and *ligustrazine hydrochloride* injection is a compound preparation composed of *ligustrazine hydrochloride* and the extract of *Salvia miltiorrhiza Bunge. Salviae miltiorrhizae Radix et Rhizoma* is a standard Chinese herbal medicine for promoting blood circulation and removing blood stasis, and is thus widely used in the treatment of cardiovascular diseases. Previous studies have shown that several active components of *Salviae miltiorrhizae Radix et Rhizoma* have significant anti-inflammatory and antioxidant activities (MEIm et al., 2019). Tanshinone IIA can reduce myocardial apoptosis and myocardial remodeling caused by overload (Feng et al., 2016) and has been shown to alleviate cardiac dysfunction in septic mice by inhibiting inflammatory response (Huang et al., 2015). Tanshinol enhances antioxidant activity by activating serine/threonine kinase/extracellular signaling-regulated kinase1/2/nuclear factor erythroid-2-related factor 2 (Akt/ERK1/2/Nrf2) signal pathway, thus exerting a cardioprotective function (Yu et al., 2015). *Ligustrazine hydrochloride* can reduce cardiomyocyte apoptosis and injury in coxsackievirus B3–induced VMC by downregulating the expression of membrane surface molecules in mouse cardiomyocytes (Jiang et al., 2014). *Breviscapine* injection, which is extracted from *Erigeron breviscapus* (*Vaniot.*) *Hand–Mazz.*, has been shown in previous studies to have pharmacological effects, such as anti-inflammation, endothelial protection, myocardial protection, and reduction of cardiac remodeling, leading to its widespread use in the treatment of cardiovascular disease (Gao et al., 2017). Shenmai injection is derived from *Panax ginseng C. A. Mey*. and *Ophiopogon japonicus* (*Thunb.*) *Ker Gawl*., and has been shown in systematic reviews to be of use in the treatment of VMC (Lu et al., 2014). Ginsenoside Rb3, an active components of *Ginseng Radix et Rhizoma*, can inhibit endothelial to mesenchymal transformation of cardiac microvascular cells following coxsackievirus B3 infection through protein-rich tyrosine kinase 2-phosphoinositide-3-kinase/serine/threonine kinase (Pyk2-PI3K-AKT) signal pathway (Yang et al., 2019). Finallly, Shenfu injection is derived from *Panax ginseng C. A. Mey* and *Aconitum carmichaeli Debeaux*, and is widely used in China for the treatment of acute and critical cardiovascular diseases. Studies have shown that Shenfu injection may play a role in antiviral myocarditis by regulating multiple metabolic pathways, such as sphingolipid metabolism, glycerophospholipid metabolism, arachidonic acid metabolism, tryptophan metabolism, amino acyl RNA biosynthesis, and the citrate cycle (Tan et al., 2018).

Although the present study compared the efficacy and safety of different types of CHIs combined with trimetazidine in the treatment of VMC by network meta-analysis and provided some reference for the selection of CHI in clinic, there are still some limitations in this study. First, the methodological quality of the included studies was generally low. The method of generating random sequences in most studies is not clear, and all studies have no random allocation hiding, which may lead to selective bias in the determination of subjects. Almost all the included studies have not blinded the patients, trial personnel, and outcome evaluator, which may lead to expectation bias due to the influence of subjective factors in the evaluation of outcome indicators. Second, there may be some clinical heterogeneity because of some differences in the age of participators, drug dosage, and course of treatment in the included studies. Some studies do not monitor safety, leading to the failure to assess the safety of some CHIs. In addition, there may be a small sample effect, resulting in some publication bias in the included study. In view of the above limitations, we suggest that more high-quality, large-sample, standardized clinical randomized controlled trials should be carried out in the future, to provide strong evidence for the efficacy and safety of CHIs combined with trimetazidine for the treatment of VMC.

## Conclusion

The results of our network meta-analysis showed that CHI combined with trimetazidine may have therapeutic effect in the treatment of VMC. Compared with conventional treatment alone or conventional treatment combined with trimetazidine, the clinical effectiveness rate of CHI combined with trimetazidine is higher, with a greater effect on reducing myocardial zymogram level and no significant difference in safety. Among these CHIs evaluated in our analysis, Shenmai injection, *Astragalus* injection, and *Salviae miltiorrhizae* and *ligustrazine hydrochloride* injection may be the most effective. Given the limitations in the design of the included studies, our conclusions require further verification in larger, multicenter, and randomized controlled trials.

## Abbreviations

VMC, viral myocarditis; CHI, Chinese herbal injection; TCM, traditional Chinese medicine; RCTs, randomized controlled trials; CNKI, China National Knowledge Infrastructure; VIP, Chinese Scientific Journals Full-Text Database; CBM, China Biology Medicine; CK, creatine kinase; CK-MB, creatine kinase-MB; LDH, lactate dehydrogenase; cTnI, cardiac troponin I; OR, odds ratios; SMD, standard mean difference; CI, confidence intervals; IF, inconsistency factors; SUCRA, surface under the cumulative ranking.
