# Supplementary material for: Evaluation of the Efficacy and Safety of Chinese Herbal Injection Combined With Trimetazidine for Viral Myocarditis: A Network Meta-Analysis
Source: Front Pharmacol. 2021 Apr 29;12:630896. doi: 10.3389/fphar.2021.630896 (PMC8117092; doi:10.3389/fphar.2021.630896)
Supplement: Supplementary file 1 [file Table1.docx]

**Supplementary Table 1.Literature search strategy**

| **Database** | **Search strategy** |
| --- | --- |
| **Pubmed** | ((((Viral myocarditis[Title/Abstract]) OR (Myocarditides[Title/Abstract]))OR (Carditis[Title/Abstract])) OR (Myocarditis[Title/Abstract])) AND ((((((((Trimetazidine[Title/Abstract]) OR (Centrophène[Title/Abstract])) OR (Trimetazidine Dihydrochloride[Title/Abstract])) OR (Dihydrochloride, Trimetazidine[Title/Abstract])) OR (Vastarel[Title/Abstract])) OR (Trimétazidine Irex[Title/Abstract])) OR (Vasartel[Title/Abstract])) OR (Idaptan[Title/Abstract])) |
| **Embase** | 1# 'viral myocarditis':ab,ti OR myocarditides:ab,ti OR carditis:ab,ti OR myocarditis:ab,ti  2# trimetazidine:ab,ti OR centrophène:ab,ti OR 'trimetazidine dihydrochloride':ab,ti OR 'dihydrochloride, trimetazidine':ab,ti OR vastarel:ab,ti OR 'trimétazidine irex':ab,ti OR vasartel:ab,ti OR idaptan:ab,ti  3# 1#AND 2# |
| **The Cochrane Library** | viral myocarditis or myocarditides or carditis or myocarditis in Title Abstract Keyword AND trimetazidine or centrophène or trimetazidine dihydrochloride or Dihydrochloride, Trimetazidine or Vastarel or Trimétazidine Irex or Vasartel or Idaptan in Title Abstract Keyword - (Word variations have been searched) |
| **CNKI** | SU=('bing du xing xin ji yan '+'xin ji yan') AND TKA=('qu mei ta qin'+'wan shuang li'+'yan suan qu mei ta qin'+'san jia yang bian qin')  (SU refers to theme;TKA refer to title,keywords and abstract.) |
| **Wanfang database** | Theme:("bing du xing xin ji yan"+"xin ji yan")*Theme:("qu mei ta qin"+"wan shuang li"+"yan suan qu mei ta qin"+"san jia yang bian qin") |
| **VIP** | (M=bing du xing xin ji yan OR M=xin ji yan) AND (M=qu mei ta qin OR M=wan shuang li OR M=yan suan qu mei ta qin OR M=san jia yang bian qin)  (M refers to title and keyword.) |
| **CBM** | [("bing du xing xin ji yan"[All fields: smart] OR "xin ji yan"[All fields: smart]) AND ( "qu mei ta qin"[All fields: smart] OR "wan shuang li "[All fields: smart] OR "yan suan qu mei ta qin"[All fields: smart] OR "san jia yang bian qin"[All fields: smart] )](http://www--sinomed--ac--cn--http.sinomed.gzzyy.qfclo.com:2222/javascript:toDoRelimitSearch();) |
