# Supplementary material for: Evaluation of the Efficacy and Safety of Chinese Herbal Injection Combined With Trimetazidine for Viral Myocarditis: A Network Meta-Analysis
Source: Front Pharmacol. 2021 Apr 29;12:630896. doi: 10.3389/fphar.2021.630896 (PMC8117092; doi:10.3389/fphar.2021.630896)
Supplement: Supplementary file 3 [file Table2.doc]

**Supplementary Table 2.Detailed information about the Chinese herbal injections used in the included studies**

| **Study** | **Chinese herbal injection** | **Source** | **Species** | **Quality control**  **reported?**  **(Y/N/U)** | **Chemical analysis**  **reported?**  **(Y/N/U)** |
| --- | --- | --- | --- | --- | --- |
| Chen,  2011 | *Astragalus* injection | Not mentioned | *Astragalus mongholicus Bunge*[Fabaceae] | U | U |
| Chen,  2014 | *Astragalus* injection | Not mentioned | *Astragalus mongholicus Bunge*[Fabaceae] | U | U |
| Dai,  2018 | *Astragalus* injection | Heilongjiang Zhenbaodao Pharmaceutical Co., Ltd. | *Astragalus mongholicus Bunge*[Fabaceae] | Y-National Food and Drug Administration National Drug Standards (Standard number:WS3-B-3335-98) | N |
| Ge,et al.,  2010 | *Astragalus* injection | Shineway Pharmaceutical Group Co., Ltd. | *Astragalus mongholicus Bunge*[Fabaceae] | Y-National Food and Drug Administration National Drug Standards (Standard number:WS3-B-3335-98) | Y-HPLC-ESI/MS |
| Ma,  2012 | *Astragalus* injection | Not mentioned | *Astragalus mongholicus Bunge*[Fabaceae] | U | U |
| Pu,  2013 | *Astragalus* injection | Not mentioned | *Astragalus mongholicus Bunge*[Fabaceae] | U | U |
| Shao,et al.,  2012 | *Astragalus* injection | Not mentioned | *Astragalus mongholicus Bunge*[Fabaceae] | U | U |
| Sun,  2013 | *Astragalus* injection | Not mentioned | *Astragalus mongholicus Bunge*[Fabaceae] | U | U |
| Wang,  2016 | *Astragalus* injection | Shineway Pharmaceutical Group Co., Ltd. | *Astragalus mongholicus Bunge*[Fabaceae] | Y-National Food and Drug Administration National Drug Standards (Standard number:WS3-B-3335-98) | Y-HPLC-ESI/MS |
| Wang,  2010 | *Astragalus* injection | Chengdu Diao Jiuhong Pharmaceutical Factory | *Astragalus mongholicus Bunge*[Fabaceae] | Y-National Food and Drug Administration National Drug Standards (Standard number:WS3-B-3335-98) | Y-Silica gel column chromatography and spectral analysis |
| Xu and Zhang,2011 | *Astragalus* injection | Chengdu Diao Jiuhong Pharmaceutical Factory | *Astragalus mongholicus Bunge*[Fabaceae] | Y-National Food and Drug Administration National Drug Standards (Standard number:WS3-B-3335-98) | Y-Silica gel column chromatography and spectral analysis |
| Yang,  2009 | *Astragalus* injection | Not mentioned | *Astragalus mongholicus Bunge*[Fabaceae] | U | U |
| Zhang,et al.,  2015 | *Astragalus* injection | Shineway Pharmaceutical Group Co., Ltd. | *Astragalus mongholicus Bunge*[Fabaceae] | Y-National Food and Drug Administration National Drug Standards (Standard number:WS3-B-3335-98) | Y-HPLC-ESI/MS |
| Yu,  2014 | *Astragalus* injection | Not mentioned | *Astragalus mongholicus Bunge*[Fabaceae] | U | U |
| Zhang,et al.,  2016 | Shenfu injection | Not mentioned | *Panax ginseng C.A.Mey.*[Araliaceae]  *Aconitum carmichaeli Debeaux*[Ranunculaceae] | U | U |
| Sun and Sun,  2018 | Shenfu injection | Not mentioned | *Panax ginseng C.A.Mey.*[Araliaceae]  *Aconitum carmichaeli Debeaux*[Ranunculaceae] | U | U |
| Gao,  2019 | Shenfu injection | China Resources Sanjiu (Ya'an)Pharmaceutical Co., Ltd. | *Panax ginseng C.A.Mey.*[Araliaceae]  *Aconitum carmichaeli Debeaux*[Ranunculaceae] | Y-National Food and Drug Administration National Drug Standards (Standard number:WS3-B-3427-98-2013） | Y-UPLC / Q-TOF-MS |
| Pang and Huang,2013 | Shenmai injection | Not mentioned | *Panax ginseng C.A.Mey.*[Araliaceae]  *Ophiopogon japonicus (Thunb.) Ker Gawl.*[Asparagaceae] | U | U |
| Wang,  2012 | *Astragalus* injection | Not mentioned | *Astragalus mongholicus Bunge*[Fabaceae] | U | U |
| Zheng,2019 | *Astragalus* injection | Shineway Pharmaceutical Group Co., Ltd. | *Astragalus mongholicus Bunge*[Fabaceae] | Y-National Food and Drug Administration National Drug Standards (Standard number:WS3-B-3335-98) | Y-HPLC-ESI/MS |
| Cui,  2018 | *Astragalus* injection | Zhengda Qingchunbao Pharmaceutical Co., Ltd. | *Astragalus mongholicus Bunge*[Fabaceae] | Y-National Food and Drug Administration National Drug Standards (Standard number:WS3-B-3335-98) | Y-LC-TOF-MS |
| Wei,  et al.,  2020 | *Astragalus* injection | Harbin Shengtai  Biopharmaceutical Co., Ltd. | *Astragalus mongholicus Bunge*[Fabaceae] | Y-National Food and Drug Administration National Drug Standards (Standard number:WS3-B-3335-98) | N |
| Zhu,  et al.,  2020 | *Salviae Miltiorrhizae* and *Ligustrazine Hydrochloride* injection | Guizhou Better Pharmaceutical Co., Ltd. | *Salvia miltiorrhiza Bunge*[Lamiaceae]  *Ligusreazine Hydrochloride* | Y-National Food and Drug Administration National Drug Standards(Standard number:WS-10001-(HD-1138)-2002-2017) | Y-UHPLC-Q-Orbitrap HRMS |
| Wang,  2020 | *Salviae Miltiorrhizae* and *Ligustrazine Hydrochloride* injection | Guizhou Better Pharmaceutical Co., Ltd. | *Salvia miltiorrhiza Bunge*[Lamiaceae]  *Ligusreazine Hydrochloride* | Y-National Food and Drug Administration National Drug Standards(Standard number:WS-10001-(HD-1138)-2002-2017) | Y-UHPLC-Q-Orbitrap HRMS |
| Chen and Zeng,  2018 | *Salviae Miltiorrhizae* and *Ligustrazine Hydrochloride* injection | Jilin Sichang Pharmaceutical Co., Ltd. | *Salvia miltiorrhiza Bunge*[Lamiaceae]  *Ligusreazine Hydrochloride* | Y-National Food and Drug Administration National Drug Standards(Standard number:WS-10001-(HD-1138)-2002-2017) | Y-HPLC |
| Li and Gao,  2016 | *Salviae Miltiorrhizae* and *Ligustrazine Hydrochloride* injection | Guizhou Better Pharmaceutical Co., Ltd. | *Salvia miltiorrhiza Bunge*[Lamiaceae]  *Ligusreazine Hydrochloride* | Y-National Food and Drug Administration National Drug Standards(Standard number:WS-10001-(HD-1138)-2002-2017) | Y-UHPLC-Q-Orbitrap HRMS |
| Miao,  2019 | *Breviscapine* injection | Yunnan Plant Pharmaceutical Co., Ltd. | *Erigeron breviscapus（Vaniot.）Hand.–Mazz.*[Asteraceae] | Y-National Food and Drug Administration National Drug Standards(Standard number:WS3-B-3822-98) | Y-UPLC-QTOF-MS |
| Wang,  2017 | *Breviscapine* injection | Shineway Pharmaceutical Group Co., Ltd. | *Erigeron breviscapus（Vaniot.）Hand.–Mazz.*[Asteraceae] | Y-National Food and Drug Administration National Drug Standards(Standard number:WS3-B-3822-98) | N |
| He,  2014 | Shenmai injection | Zhengda Qingchunbao Pharmaceutical Co., Ltd. | *Panax ginseng C.A.Mey.*[Araliaceae]  *Ophiopogon japonicus (Thunb.) Ker Gawl.*[Asparagaceae] | Y-National Food and Drug Administration National Drug Standards(Standard number:WS3-B-3428-98-2010) | Y-HPLC-MS/MS |

Note:Y-yes;N-no;U:uncertain

**Supplementary Table 3.Chemical analysis of five types of Chinese herbal injections**

| **Chinese herbal injections** | **Source** | **Quality control**  **reported?**  **(Y/N)** | **Chemical**  **analysis**  **reported?**  **(Y/N)** | **Species/Raw materials ratio** | **Chemical composition** | **Chemical composition standard** |
| --- | --- | --- | --- | --- | --- | --- |
| *Astragalus* injection | Shineway Pharmaceutical Group Co., Ltd./ | Y-National Food and Drug Administration National Drug Standards (Standard number:WS3-B-3335-98) | Y-HPLC-ESI/MS | *Astragalus mongholicus Bunge*[Fabaceae] | AstragalosideIV, Astragaloside I ,  AstragalosideV,  AstragalosideVI,  AstragalosideVII,  Isorhamnetin ,  Astragalin,  Pratensein(P), Pratensein 7-O-β-D-  glucopyranoside,  Medicarpin(P),  and et al | Each 1ml *Astragalus* injection contains Astragaloside IV (C41H68O14) not less than 0.08mg |
| Shenfu injection | China Resources Sanjiu (Ya'an)Pharmaceutical Co., Ltd. | Y-National Food and Drug Administration National Drug Standards (Standard number:WS3-B-3427-98-2013） | Y-UPLC/ Q-TOF-MS | *Panax ginseng C.A.Mey.*[Araliaceae]  and *Aconitum carmichaeli Debeaux*[Ranunculaceae]**/**  *Ginseng Radix et Rhizoma Rubra*：*Aconiti Lateralis Radix Praeparata*=1:2 | GinsenosideRb1,  GinsenosideRb2,  GinsenosideRa1,  GinsenosideRa2,  Aconitine,  mesaconitine  and et al | Each 1ml Shenfu injection contains aconitine alkaloids not more than 0.1mg, calculated as Aconitine (C24H47NO11);  Each 1ml Shenfu injection contains Ginseng total saponin not less than 0.5mg, based on Ginseng soap Rb1 (C54H92O23). |
| *Salviae Miltiorrhizae* and *Ligustrazine Hydrochloride* injection | Jilin Sichang Pharmaceutical Co., Ltd./ | Y-National Food and Drug Administration National Drug Standards(Standard number:WS-10001-(HD-1138)-2002-2017) | Y-HPLC | *Salvia miltiorrhiza Bunge*[Lamiaceae] and  *Ligusreazine Hydrochloride***/**  *Salviae Miltiorrhizae Radix Et Rhizoma:Ligusreazine Hydrochlorid*e=10:1 | Danshensu Sodium ,  *Ligustrazine Hydrochloride* ,  Isoferulic acid,  Rosmarinic acid,  Salvianolic acid A  and et al | Each 1ml of *Salviae Miltiorrhizae* and *Ligustrazine Hydrochloride* injection should contain Danshensu(C9H10O5) 0.36-0.44mg |
| *Breviscapine* injection | Yunnan Plant Pharmaceutical Co., Ltd./ | Y-National Food and Drug Administration National Drug Standards(Standard number:WS3-B-3822-98) | Y-UPLC-QTOF-MS | *Erigeron breviscapus（Vaniot.）Hand.–Mazz.*[Asteraceae] | Scutellarin ,and et al | The amount of Scutellarin  (C21H18O12) in *Breviscapine* injection should be 95.0-105.0% of the labeled amount |
| Shenmai injection | Zhengda Qingchunbao Pharmaceutical Co., Ltd. | Y-National Food and Drug Administration National Drug Standards(Standard number:WS3-B-3428-98-2010) | Y-HPLC-MS/MS | *Panax ginseng C.A.Mey.*[Araliaceae] and  *Ophiopogon japonicus (Thunb.) Ker Gawl.*[Asparagaceae]**/**  *Ginseng Radix et Rhizoma Rubra:*  *Ophiopogonis Radix*  =1:1 | GinsenosideRg1,  GinsenosideRe,  GinsenosideRb1, Ophiopogonin D,  Ophiopogonin D′,  methylophiopogonanone A,  methylophiopogonanone B and et al | Each 1ml Shenmai injection should contain the total saponins 0.80-2.00mg,based on ginsenoside Re (C48H82O18). |

**References**

Zhang, G.,Hu, X.J.,Jiang, G.Z.,Liu, Y.L.,Ba, X.Y.(2016).Identification of small molecular organic compounds of astragalus injection by LC/MS.*Mod Chin Med,*18(4),410-414,430.https://doi.org/10.13313/j.issn.1673-4890.2016.4.004

Dou, W.,Fu, T.J.,Zhang, F.,Liu, Z.R.,Ding, L.S.(2002).Chemical constituents of astragalus injection.*Natural Product Research and Development,*14(6),14-17.http://doi.org/10.16333/j.1001-6880.2002.06.005

Song, H.T.,Li, C.Y.,Wang, Y.Y.,Ding, X.S.,Tan, X.Y.,Dai, G.L.,Liu, S.J.,Ju, W.Z.(2017).Screen astragalosides from Huangqi injections by LC-TOF-MS-based mass defect filtering approach.*China journal of Chinese materia medica,*42(4):686-695.http://doi.org/10.19540/j.cnki.cjcmm.20170103.017

He, J.L.,Zhou, S.S.,Ma, Z.C.,Liang, Q.D.,Wang, Y.G.,Tan, H.L.,Xiao, C.R.,Tang, X.L.,Gao, Y.(2014).Material basis of Shenfu injection based on UPLC-Q-TOF / MS.*Chinese Pharmacological Bulletin*,30(3):429-433.http://doi.org/10.3969 / j.issn.1001-1978.2014.03.029

Zhou, P.P.,Zhou, L.,Sun, Z.,Li, Z.L.,Zhang, R.,Guan, K.L.,Yang, Y.J.,Li, H.M.,Wang, Y.H.,Du, X.Y.,Zhang, X.L.,Du, S.Z.(2019).Qualitative and Quantitative Analysis of Danshen-chuanxiongqin Injection by Using UHPLC-Q-Orbitrap HRMS.*Chin Pharm J*,54(4):327-333.http://doi.org/10. 11669 / cpj. 2019. 04. 012

Dong, Q.H.,Wu, F.L.,Wang, H.,Tan, J.,Lin, H.Q.,Liu, J.P.,Li, P.Y.(2018).HPLC Fingerprint Study of Danshen Chuanxiongqin Injection and Its Salvia miltiorrhiza Extract.*Journal of Chinese Medicinal Materials,*41(11),2611-2614.https://doi.org/10. 13863 / j. issn1001-4454. 2018. 11. 026

Zhao, M., Wang, P. F., Wang, X. M., Liu, Y., Liu, X. Q., Chen, L. M., Gao, H. M., Wang, Z. M., & Zhang, W. (2018).Structural identification of related substances in Breviscapine by UPLC-QTOF-MS.*China journal of Chinese materia medica*, 43(14), 2872-2877. <https://doi.org/10.19540/j.cnki.cjcmm.2018.0089>

Wu, Y.,Wei, X.,Zhang, L.Y.,Yu, Z.F.,Ren, B.N.,Qi, J.L.,Dong, Z.J.(2014).Simultaneous determination of seven components in Shenmai Injection by HPLC-MS/MS.*Chinese Traditional and Herbal Drugs*,45(18),2625-2630.https://doi.org/10.7501/j.issn.0253-2670.2014.18.010
